# Power-law scaling to assist with key challenges in artificial intelligence

**DOI:** 10.1038/s41598-020-76764-1

**Published:** 2020-11-12

**Authors:** Yuval Meir, Shira Sardi, Shiri Hodassman, Karin Kisos, Itamar Ben-Noam, Amir Goldental, Ido Kanter

**Affiliations:** 1 Department of Physics, Bar-Ilan University, 52900 Ramat-Gan, Israel; 2 Gonda Interdisciplinary Brain Research Center, Bar-Ilan University, 52900 Ramat-Gan, Israel

**Keywords:** Information theory and computation, Statistical physics, thermodynamics and nonlinear dynamics

## Abstract

Power-law scaling, a central concept in critical phenomena, is found to be useful in deep learning, where optimized test errors on handwritten digit examples converge as a power-law to zero with database size. For rapid decision making with one training epoch, each example is presented only once to the trained network, the power-law exponent increased with the number of hidden layers. For the largest dataset, the obtained test error was estimated to be in the proximity of state-of-the-art algorithms for large epoch numbers. Power-law scaling assists with key challenges found in current artificial intelligence applications and facilitates an a priori dataset size estimation to achieve a desired test accuracy. It establishes a benchmark for measuring training complexity and a quantitative hierarchy of machine learning tasks and algorithms.

## Introduction

Phase transition and critical phenomena have been the central focus of statistical mechanics, since the beginning of the second half of twentieth century. The thermodynamic properties near the critical point of second-order phase transitions were explained using power-law scaling and hyperscaling relations, depending on the dimensionality of the system^1,2^. The concept of power-law implies a linear relationship between the logarithms of two quantities, that is, a straight line on a log–log plot. It arises from diverse phenomena including the timing and magnitude of earthquakes^3^, internet topology and social networks^4–6^, turbulence^7^, stock price fluctuations^8^, word frequencies in linguistics^9^ and signal amplitudes in brain activity^10^.

**Figure 1:**
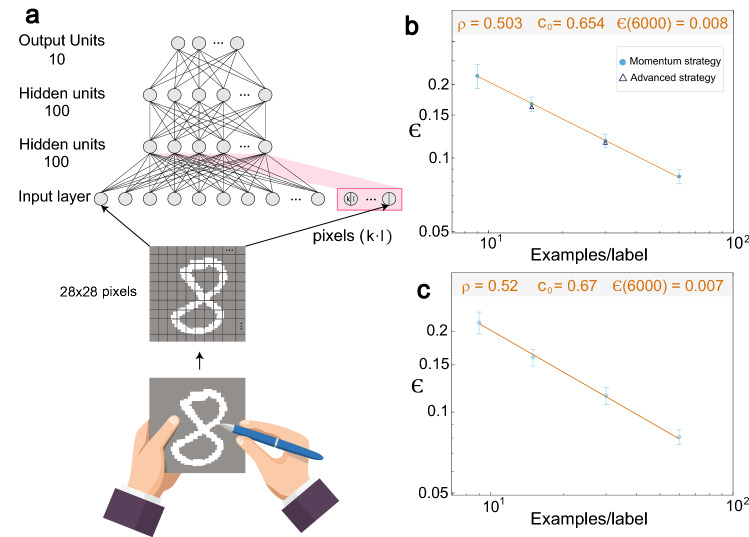
Power-law scaling for the test error with many epochs. (**a**) Scheme of MNIST handwritten digit, which is digitized and fed into the trained network including input crosses (red background). (**b**) Optimized test error, $\epsilon ,$ using the architecture in (**a**), for limited datasets comprising 9, 15, 30 and 60 examples/label and their standard deviations obtained from 50 samples. Momentum strategy (light-blue circles) and advanced, i.e. accelerated, strategy (black triangles). (**c**) Test error for soft committee decision with $N_{c} = 50$ (Eq. 8). (For details of the parameters, see Supplementary Appendix B).

Deep learning algorithms are found to be useful in an ever-increasing number of applications, including the analysis of experimental data in physics, ranging from classification problems in astrophysics^11^ and high-energy physics data analysis^12^ to imaging in noise optics^13^ and learning properties of phase transitions^14^. This work indicates that deep learning algorithms behave asymptotically similar to critical physical systems. A basic task in deep learning is supervised learning, where a multilayer network (e.g. Fig. 1a) learns to produce the correct output labels to the input data based on a training database of examples, input–output pairs. A simple example of this is the large Modified National Institute of Standards and Technology (MNIST) database consisting of 60,000 training handwritten digits and 10,000 test digits^15^, without any data extension^16,17^. The weights of the selected feedforward network are adjusted using back-propagation algorithm, which is a gradient-descent-based algorithm, to minimize the cost function, thereby, quantifying the mismatch between the current and desired outputs^15^.

The performance of the algorithm is estimated using test error, measured on a dataset that was not observed during the training. The test error is expected to decrease with increasing information and increasing dataset size, and to vanish asymptotically in a sufficiently complex network, e.g. enough number of weights, hidden layers and units. The disappearance of the test error with a power-law scaling is the focus of our study, which sets a priori estimation of the required dataset size to achieve the desired test accuracies. The robustness of the power-law scaling phenomenon is examined for training with one and many epochs, that is, for the number of times each example is presented to the trained network, as well as for several feedforward network architectures consisting of a few hidden layers and hyper-weights^18^, that is, input crosses. The result of the optimized test errors with one training epoch is in the proximity of state-of-the-art algorithms consisting of a large number of epochs, which has an important implication on the rapid decision making under limited numbers of examples^19,20^, which is representative of many aspects of human activity, robotic control^21^, and network optimization^22^. The current applicability of the asymptotic test accuracy to such realities using an extremely large number of epochs is questionable. This large gap between advanced learning algorithms and their real-time implementation can be addressed by achieving optimal performance based on only one epoch. Finally, the comparison of the power-law scaling, exponents and constant factors, stem from various learning tasks, datasets, and algorithms is expected to establish a benchmark for a quantitative theoretical framework to measure their complexity^23^.

The first trained network that is employed comprises 784 inputs representing 28 × 28 pixels of a handwritten digit in the range [0, 255] with additional 10,000 input crosses per hidden unit (see Supplementary Appendix A), two hidden layers comprising 100 units each, and 10 outputs representing the labels (Fig. 1a). The presented dataset of examples for the algorithm involves the following initial preprocessing and steps (see Supplementary Appendix A): (a) *Balanced set of examples*: The small dataset consists of an equal number of random examples per label^24^. (b) *Input bias:* The bias of each example is subtracted and the standard deviation of its 784 pixels is normalized to unity. (c) *Fixed order of trained labels*: In each epoch, examples are ordered at random, conditioned to the fixed order of the labels. (d) *Microcanonical set of input crosses:* Each hidden unit in the first layer receives the same number of input crosses, in which each cross comprises two input pixels. (e) *Forward propagation:* A standard sigmoid activation function is attributed to each node^25^ and in the forward propagation the accumulative average field is dynamically subtracted from the induced field on each node of the hidden layers.

## Results

### Momentum strategy: power-law with many epochs

The commonly used learning approach is the backpropagation (BP) strategy given by:

1 $$W^{t + 1} = W^{t} - \eta \cdot \nabla_{{W^{t} }} C { }$$

where a weight at discrete time-step t, W^t^, is modified with a step-size η towards the minus sign of the gradient of the cross entropy cost function, C,

2 $${\text{C}} = - \frac{1}{{\text{M}}}\mathop \sum \limits_{{{\text{m}} = 1}}^{{\text{M}}} \left[ {y_{m} \cdot \log \left( {a_{m}^{L} } \right) + \left( {1 - y_{m} } \right) \cdot \log \left( {1 - a_{m}^{L} } \right)} \right] + \frac{\alpha }{2\eta }\mathop \sum \limits_{i} W_{i }^{2}$$

where *y*_*m*_ stands for the desired labels of the m^th^ examples, $a_{m}^{L}$ stands for the current 10 outputs of the output layer L, and the first summation is over all M training examples. The second summation is the overall weights of the network, and $\eta$ and $\alpha$ are constants defined in Eqs. (1) and (3), respectively. Here we used the momentum strategy^26^:

3 $$\begin{aligned} V^{t + 1} & = \mu \cdot V^{t} - \eta \cdot \nabla_{{W^{t} }} C \\ W^{t + 1} & = \left( {1 - \alpha } \right) \cdot W^{t} + V^{t + 1} \\ \end{aligned}$$

where the friction, μ, and the regularization of the weights, 1-α, are global constants in the region [0, 1] and ${\upeta }$ is a constant representing the learning rate. In addition there are biases per node associated with the induced field on each node

4 $$\begin{aligned} {\text{V}}_{{\text{b}}}^{{{\text{t}} + 1}} & = {\upmu } \cdot {\text{V}}_{{\text{b}}}^{{\text{t}}} - \eta \cdot \nabla_{{{\text{b}}^{{\text{t}}} }} {\text{C }} \\ {\text{b}}^{{{\text{t}} + 1}} & = {\text{b}}^{{\text{t}}} + {\text{V}}_{{\text{b}}}^{{{\text{t}} + 1}} { } \\ \end{aligned}$$

We minimize the test error for each dataset size over the five parameters of the algorithm ($\mu , \alpha , \eta , Amp_{1} , Amp_{2} )$ (where Amp_i_ are the amplitudes associated with each hidden layer in the forward propagation, see Supplementary Appendix A). The minimized averaged test error, $\epsilon$, for number of examples per label in the range [9,120] indicates a power-law scaling

5 $$\epsilon \sim \frac{{c_{0} }}{{\left( {dataset\, size/label} \right)^{\rho } }}$$

with $c_{0} \sim 0.65,$ $\rho \sim 0.50$ (Fig. 1b), and its extrapolation to the maximal dataset, 6,000 examples per label, indicates a test error of $\epsilon \sim 0.008$. Note that the saturation of the minimal test error is achieved after at least 150 epochs (see Supplementary Appendix B).

### Accelerated strategy: power-law with many epochs

An accelerated BP method is based on a recent new bridge between experimental neuroscience and advanced artificial intelligence learning algorithms, in which an increased training frequency has been able to significantly accelerate neuronal adaptation processes^24^. This *accelerated* brain-inspired mechanism involves time-dependent step-size, ${\upeta }^{{\text{t}}}$, associated with each weight, such that coherent consecutive gradients of weight, that is, with the same sign, increase the conjugate ${\upeta }$. The discrete time BP of this accelerated method is summarized for each weight by

6 $$\begin{aligned} {{ \upeta }}^{{{\text{t}} + 1}} & = {\upeta }^{{\text{t}}} \cdot {\text{e}}^{{ - {\uptau }}} + {\text{A}} \cdot {\text{tanh}}\left( {\beta \cdot \nabla_{{{\text{W}}^{{\text{t}}} }} {\text{C}}} \right) \\ {\text{V}}^{{{\text{t}} + 1}} & = {\upmu } \cdot {\text{V}}^{{\text{t}}} - |{\upeta }^{{{\text{t}} + 1}} | \cdot \nabla_{{{\text{W}}^{{\text{t}}} }} {\text{C}} \\ {\text{W}}^{{{\text{t}} + 1}} & = \left( {1 - {\upalpha }} \right) \cdot {\text{W}}^{{\text{t}}} + {\text{V}}^{{{\text{t}} + 1}} \\ \end{aligned}$$

where A and β are constants, different for each layer, representing the amplitude and gain, respectively. In addition, there are biases per node similar to Eq. (4) where $\eta_{0}$ is replaced by time-dependent $\eta_{b}^{t}$

7 $$\begin{aligned} {{ \upeta }}_{{\text{b}}}^{{{\text{t}} + 1}} & = {\upeta }_{{\text{b}}}^{{\text{t}}} \cdot {\text{e}}^{{ - {\uptau }}} + {\text{A}} \cdot {\text{tanh}}\left( {\beta \cdot \nabla_{{{\text{b}}^{{\text{t}}} }} {\text{C}}} \right) \\ {\text{V}}_{{\text{b}}}^{{{\text{t}} + 1}} & = {\upmu } \cdot {\text{V}}_{{\text{b}}}^{{\text{t}}} - |{\upeta }_{{\text{b}}}^{{{\text{t}} + 1}} | \cdot \nabla_{{{\text{b}}^{{\text{t}}} }} {\text{C}} \\ {\text{ b}}^{{{\text{t}} + 1}} & = {\text{b}}^{{\text{t}}} + {\text{V}}_{{\text{b}}}^{{{\text{t}} + 1}} \\ \end{aligned}$$

The minimization of the test error of this accelerated method over its 11 parameters $(A_{1} ,A_{2} , A_{3} , \beta_{1} ,\beta_{2} , \beta_{3} , \mu ,\alpha , \tau , Amp_{1} , Amp_{2} )$ (see Supplementary Appendix A) is a computational heavy task. It results in the same saturated test error as that for the momentum strategy (Fig. 1b), however, with only 30–50 epochs owing to its accelerated nature.

The test error is further minimized using a soft committee decision based on several replicas, *Nc*, of the network, which are trained on the same set of examples but with different initial weights. The result label, j, for the test accuracy is given by

8 $$\mathop {\max }\limits_{j} \left( {\mathop \sum \limits_{s = 1}^{{N_{c} }} a_{{{\text{j}},{\text{s}}}}^{{\text{L}}} } \right)$$

where $a_{j,s}^{L}$ stands for the value of the output label j in output layer L and in replica s (j = 0, 1, …0.9). The minimized test error of the soft committee of the momentum strategy is $\epsilon \sim 0.007$ with $\rho \sim 0.52$(Fig. 1c), which is in close agreement with state-of-the-art achievements obtained using deep neural networks^27^.

### Power-law with one epoch

**Figure 2:**
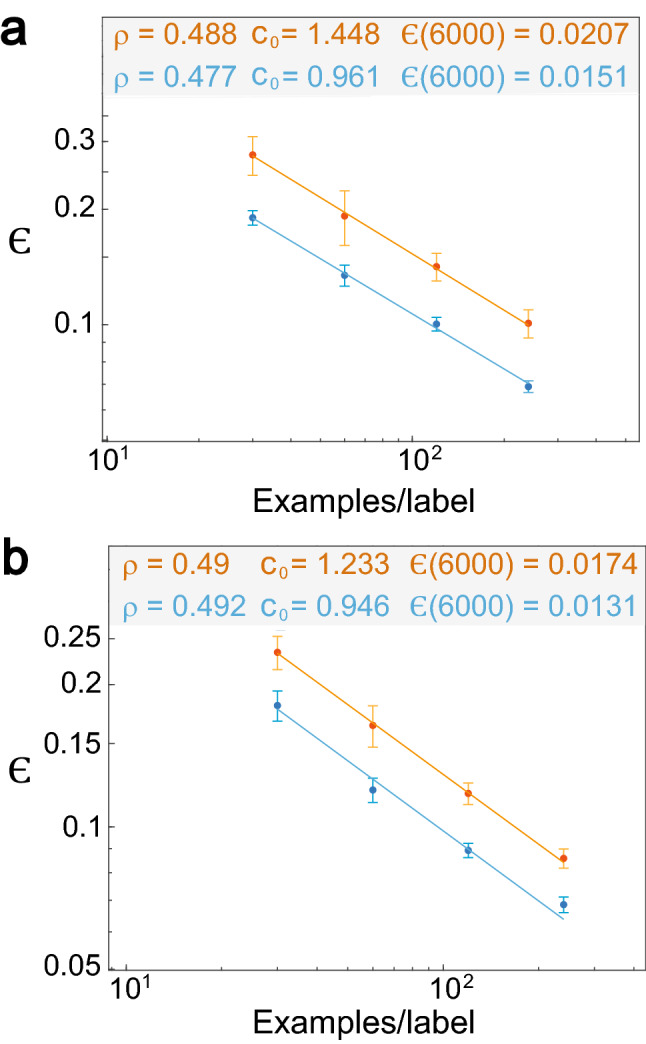
Power-law scaling for the test error with one epoch. (**a**) Test error and its standard deviation as a function of number of examples per label for one epoch only where the trained network is the same as in Fig. 1a. Results for the momentum strategy (orange) and for the soft committee, $N_{c} = 50$, (blue), where each point is averaged over at least 100 samples. (**b**) Similar to (**a**) using the accelerated BP strategy, Eqs. (6) and (7). (For details of the parameters, see Supplementary Appendix C).

A similar minimization of the test error, $\epsilon ,$ is repeated for one epoch, where each example in the training set is presented only once as an input to the feedforward network (Fig. 1a). For the momentum strategy it is found that $\rho \sim 0.49$ and its extrapolation to the maximal dataset (i.e., 6,000 examples per label) results in $\epsilon \sim 0.021$ (Fig. 2a), and for the brain-inspired accelerated strategy in $\epsilon \sim 0.017$ and $\rho \sim 0.49$ (Fig. 2b). For the soft committee of the momentum strategy it is found that $\epsilon \sim 0.015$ with slope, $\rho \sim 0.48$ (Fig. 2a). The test error is reduced even further using soft committee of the accelerated strategy, where $\epsilon \sim 0.013$ with slope, $\rho \sim 0.49$ for 6,000 examples per label (Fig. 2b). Results of one epoch are in the proximity of the test error using many epochs, where the best test error for many epochs $\epsilon \sim 0.007$ has to be compared with $\epsilon \sim 0.013$ for one epoch. These results strongly indicate that rapid decision making, which is representative of many aspects of human activity, robotic control^28^, and network optimization^22^, is feasible.

### Power-law with several hidden layers

**Figure 3:**
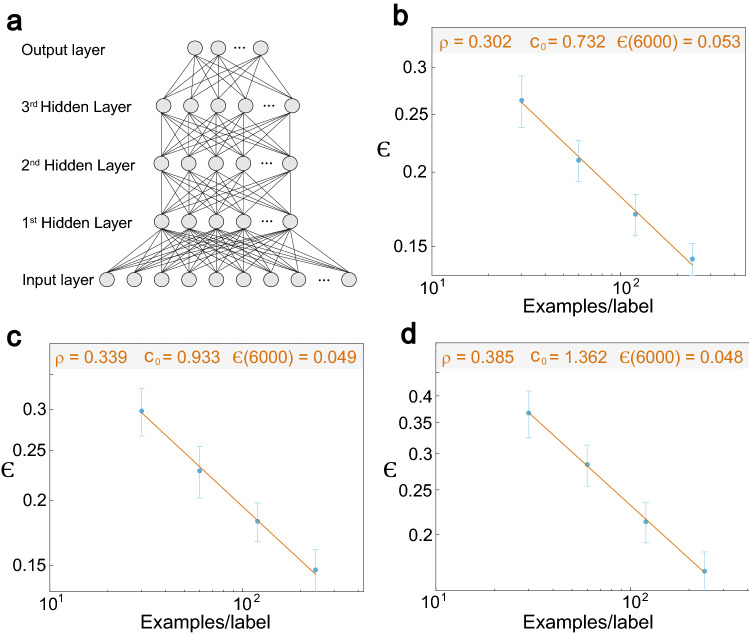
Power-law scaling for the test error with several hidden layers and one epoch. (**a**) Scheme of the trained network on the MNIST examples consisting of three hidden layers having each 100 units and an output layer. In the case of one/two hidden layers only, two/one hidden layers are removed. (**b**) Minimized test error for 30, 60, 120, and 240 examples/label for one hidden layer (**a**) using the momentum strategy and one epoch only. The average of each point and its standard deviation are obtained from at least 100 samples. (**c**) Similar to (**b**) with two hidden layers in (**a**). (**d**) Similar to (**b**) with three hidden layers in (**a**). (For details of the parameters, see Supplementary Appendix D).

**Figure 4:**
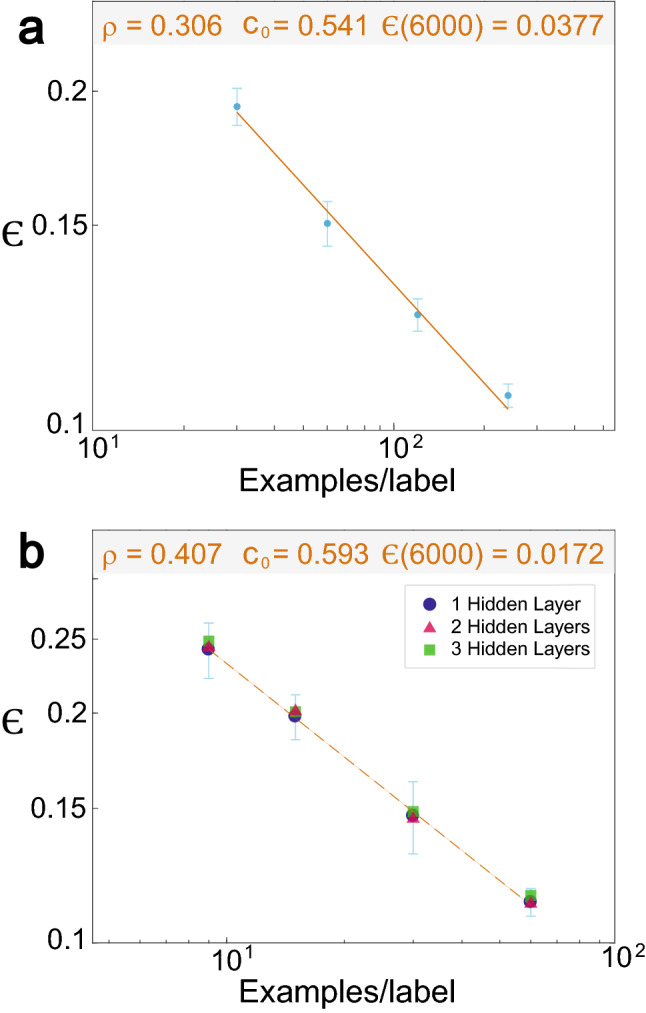
(**a**) Test error, *ε*, as a function of the number of examples per label for soft committee decision (N_c_ = 50 in Eq. 8), for two hidden layers without input crosses and one epoch, presented in Fig. 3c. (**b**) Saturated test error obtained for many epochs as a function of the number of examples per label, for the feedforward network (Fig. 3a), one hidden layer (light-blue circles), two hidden units (orange triangles), and three hidden units (green squares). Typical error bars obtained from at least 200 samples for each number of examples per labels are presented. (For details of the parameters, see Supplementary Appendix E).

The robustness of the power-law phenomenon for the test error as a function of dataset size (Figs. 1, 2) is examined for similar feedforward networks without input crosses, and with up to three hidden layers with 100 hidden units each (Fig. 3a). For one hidden layer, the minimization of $\epsilon$ for one epoch and for the momentum strategy indicates $\rho \sim 0.3,$ and its extrapolation to 6,000 examples per label results in $\epsilon = 0.053$ (Fig. 3b). Using two layers the exponent increases to $\rho \sim 0.34$ with $\epsilon = 0.049$ (Fig. 3c), and for three layers to $\rho = 0.385$ with $\epsilon = 0.048$ (Fig. 3d). These results confirm the existence of the power-law phenomenon in a larger class of feedforward networks and different learning rules as well as the possible increase of the power-law exponent with increasing number of hidden layers (Fig. 3b–d). Asymptotically for very large datasets, increasing the number of hidden layers is expected to minimize $\epsilon$, since $\rho$ increases. However, for a limited number of examples, one layer minimizes $\epsilon$ (Fig. 3b–d), as the constant $c_{0}$ in Eq. (5) is smaller for one layer. Particularly, the power-law scaling indicates that the crossing of $\epsilon$ between one and two layers occurs at $\sim 480$ examples per label, whereas the crossing between two and three layers occurs at $\sim 4100$ examples per label. This trend stems from the limit of small training datasets and one training epoch, which prevents enhanced optimization of the many more weights of networks with more hidden layers. The asymptotic test error, $\epsilon = 0.049,$ of a network with two hidden layers (Fig. 3c) has to be compared with $\epsilon \sim 0.021$ which is achieved for the same architecture with additional input crosses (Fig. 2a). The significant improvement of $\sim 0.028$ is attributed to the additional input crosses. This gap also remains under soft committee decision where for two layers without input crosses and the maximal dataset, 6,000 examples per label, $\epsilon \sim 0.038$ (Fig. 4a), which is much greater than $\epsilon \sim 0.015$ (Fig. 2a). We note that $\rho \sim 0.31$ (Fig. 4a) is expected to slightly increase beyond $\rho \sim 0.34$ (Fig. 3c) using better statistics.

## Discussion

The power-law scaling enables the building of an initial step for theoretical framework for deep learning by feedforward neural networks. A classification task, which is characterized by a much smaller power-law exponent, $\rho ,$ is categorized as a much harder classification problem. It demands a much larger dataset size to achieve the same test error, as long as the constant $c_{0}$ (Eq. (5)) is similar. Similarly, one can compare the efficiency of optimal learning strategy by two different architectures for the same dataset and number of epochs (Figs. 2, 3) or a comparison of two different BP strategies for the same architecture (Fig. 1). Our work calls for the extension and the confirmation of the power-law scaling phenomenon in other datasets^23,29–32^, which will enable to build a hierarchy among their learning complexities. It is especially interesting to observe whether the power-law scaling will lead to a test error in the proximity of state-of-the-art algorithms for other classification and decision problems as well.

The observation in which the test error with one training epoch is in the proximity of the minimized test error using a very large number of epochs paves way for the realization of deep learning algorithms in real-time environments, such as tasks in robotics and network control. A relatively small test error, for instance less than 0.1, can be achieved for a small datasets consisting of only a few tens of examples per label only.

Finally, under the momentum strategy and many training epochs, the minimal saturated test errors of one, two, and three hidden layers and without input crosses are found to be very similar (Fig. 4b). The test error, $\epsilon \sim 0.017$, at the maximal dataset size and *ρ* ~ 0.4 has to be compared to $0.008$ with additional input crosses and $\rho \sim 0.5$ (Fig. 1b). For three layers, $\epsilon$ is slightly greater than for one or two layers, but within the error bars. This gap diminishes when the optimized test error for the three layers is obtained under an increased number of epochs, and through the construction of weighs one can show that $\epsilon$ of two layers is achievable with three layers(see Supplementary Appendix F). Furthermore, the similarity of $\epsilon$, independent of the number of hidden layers and for many training epochs (Fig. 4b), is supported by our preliminary results, wherein the average $\epsilon$ of one hidden layer with input crosses and many training epochs is comparable with the one obtained with two hidden layers (Fig. 1b). These results may question the advantage of deep learning based on many hidden layers in comparison to shallow architectures. It is possible that this similarity in the test errors, independent of the number of hidden layers, is either an exceptional case or a larger number of hidden layers enables an easier search in the BP parameters space, which achieves proximity solutions of the minimal test error. However, for the same examined architectures and for one epoch only, the test error and the exponent of the power-law are strongly dependent on the number of hidden layers (Fig. 3).

## Supplementary information

Supplementary Information.

## References

[1] Wilson KG (1975). The renormalization group: critical phenomena and the Kondo problem. Rev. Mod. Phys..

[2] Ma S (1976). Modern Theory of Critical Phenomena.

[3] Bak P, Christensen K, Danon L, Scanlon T (2002). Unified scaling law for earthquakes. Phys. Rev. Lett..

[4] Song C, Havlin S, Makse HA (2005). Self-similarity of complex networks. Nature.

[5] Albert R, Barabási A-L (2002). Statistical mechanics of complex networks. Rev. Mod. Phys..

[6] Adamic LA (2000). Power-law distribution of the world wide web. Science.

[7] She Z-S, Leveque E (1994). Universal scaling laws in fully developed turbulence. Phys. Rev. Lett..

[8] Gabaix X (2009). Power laws in economics and finance. Annu. Rev. Econ..

[9] Kanter I, Kessler D (1995). Markov processes: linguistics and Zipf's law. Phys. Rev. Lett..

[10] Miller KJ, Sorensen LB, Ojemann JG, Den Nijs M (2009). Power-law scaling in the brain surface electric potential. PLoS Comput. Biol..

[11] Huerta EA (2019). Enabling real-time multi-messenger astrophysics discoveries with deep learning. Nat. Rev. Phys..

[12] Guest D, Cranmer K, Whiteson D (2018). Deep learning and its application to LHC physics. Annu. Rev. Nucl. Part. Sci..

[13] Goy A, Arthur K, Li S, Barbastathis G (2018). Low photon count phase retrieval using deep learning. Phys. Rev. Lett..

[14] Wang L (2016). Discovering phase transitions with unsupervised learning. Phys. Rev. B.

[15] LeCun Y (1995). Learning algorithms for classification: a comparison on handwritten digit recognition. Neural Netw. Stat. Mech. Perspect..

[16] Zhang Y, Ling C (2018). A strategy to apply machine learning to small datasets in materials science. NPJ Comput. Mater..

[17] Hoffmann J (2019). Machine learning in a data-limited regime: augmenting experiments with synthetic data uncovers order in crumpled sheets. Sci. Adv..

[18] Buldyrev SV, Parshani R, Paul G, Stanley HE, Havlin S (2010). Catastrophic cascade of failures in interdependent networks. Nature.

[19] D’souza RN, Huang P-Y, Yeh F-C (2020). Structural analysis and optimization of convolutional neural networks with a small sample size. Sci. Rep..

[20] Delahunt CB, Kutz JN (2019). Putting a bug in ML: the moth olfactory network learns to read MNIST. Neural Netw..

[21] Edelman, B. J. *et al.* Noninvasive neuroimaging enhances continuous neural tracking for robotic device control. *Sci. Robot. 4* (2019). 10.1126/scirobotics.aaw6844 PMC6814169 31656937

[22] Mateo D, Horsevad N, Hassani V, Chamanbaz M, Bouffanais R (2019). Optimal network topology for responsive collective behavior. Sci. Adv..

[23] Rosenfeld, J. S., Rosenfeld, A., Belinkov, Y. & Shavit, N. A constructive prediction of the generalization error across scales. arXiv preprint arXiv:1909.12673 (2019).

[24] Sardi S (2020). Brain experiments imply adaptation mechanisms which outperform common AI learning algorithms. Sci. Rep..

[25] Narayan S (1997). The generalized sigmoid activation function: competitive supervised learning. Inf. Sci..

[26] Rumelhart DE, Hinton GE, Williams RJ (1986). Learning representations by back-propagating errors. Nature.

[27] Kowsari, K., Heidarysafa, M., Brown, D. E., Meimandi, K. J. & Barnes, L. E. in *Proceedings of the 2nd International Conference on Information System and Data Mining.* 19–28.

[28] Edelman B (2019). Noninvasive neuroimaging enhances continuous neural tracking for robotic device control. Sci. Robot..

[29] Krizhevsky, A. & Hinton, G. Learning multiple layers of features from tiny images (2009).

[30] Russakovsky O (2015). Imagenet large scale visual recognition challenge. Int. J. Comput. Vis..

[31] Fei-Fei, L., Fergus, R. & Perona, P. in *2004 conference on computer vision and pattern recognition workshop.* 178–178 (IEEE).

[32] Hestness, J. *et al.* Deep learning scaling is predictable, empirically. arXiv preprint arXiv:1712.00409 (2017).

