# Supplementary material for: Power-law scaling to assist with key challenges in artificial intelligence
Source: Sci Rep. 2020 Nov 12;10:19628. doi: 10.1038/s41598-020-76764-1 (PMC7665018; doi:10.1038/s41598-020-76764-1)
Supplement: Supplementary file 1 — Supplementary Information. [file 41598_2020_76764_MOESM1_ESM.pdf]

Supplementary Information

Power-law Scaling to Assist with Key Challenges in Artificial Intelligence

Yuval Meir[1,*] , Shira Sardi[1,*], Shiri Hodassman[1,*], Karin Kisos[1], Itamar Ben-Noam[1], Amir Goldental[1] & Ido Kanter[1,2,†]

[1]Department of Physics, Bar-Ilan University, Ramat-Gan, 52900, Israel.

[2]Gonda Interdisciplinary Brain Research Center, Bar-Ilan University, Ramat-Gan, 52900, Israel.

[*] These authors contributed equally to this work

[†]Corresponding author: ido.kanter@biu.ac.il (I.K.)

APPENDIX A: DETAILS OF THE USED ALGORITHM

Architecture and initial weights: The feedforward neural network (Fig. 1) consists of 784 input units with additional 10,000 input-crosses for each hidden unit (see Input), 2 hidden layers consist of 100 units each and 10 output units. Weights between successive layers are fully connected, except the input-crosses. Each unit in the hidden and the output layers has an additional input from a bias unit. We denote by W^1, W^2 and W^3 the weights from the input layer to the first hidden layer, from the first hidden layer to the second hidden one and from the second hidden layer to the output layer, respectively. The initial conditions of all weights are randomly chosen from a Gaussian distribution with a zero average and standard deviation (STD) equals 1. All weights are normalized such that all input weights to each hidden unit have a zero average and an STD equals 1[1].

After the above-mentioned initial normalization of all weights, the weights of the input-crosses are rescaled

$$W_{input\ crosses} = \sqrt{\frac{\#\ regular\ input}{\#\ input\ crosses}} \cdot W_{input\ crosses} = \sqrt{\frac{784}{10000}} \cdot W_{input\ crosses}$$

such that initially they have the same effect on the forward propagation as the regular weights. In addition, the initial value of the bias of each weights is set to 1.

Input: Each example, $\tilde{X}_m, m = 1, 2, \dots, M$, of the train dataset consists of 784 pixels, $\tilde{X}_{m,p}$, which their values are in the range [0, 255]. The input, X, of the example $\tilde{X}$, consists of the original 784 pixels where the average pixel value in $\tilde{X}$ is subtracted from each pixel and the standard deviation is set to one:

$$\mathrm{X_m} = \tilde{X}_m - \frac{1}{784}\sum_{\mathrm{p}=1}^{784} \tilde{X}_{m,p}$$

$$\mathrm{X_m} = \mathrm{X_m}/std\left(\tilde{X}_m\right)$$

Furthermore, an input pixel which has an identical value among all the training examples, e.g. have zero variance in all train dataset examples, is set to zero.

An addition of 10,000 input-crosses are added to the input, $\mathrm{X_{k,l}}$:

$$\mathrm{X_{k,l}} = \mathrm{X_k} \cdot \mathrm{X_l}$$

where k and l are random indices in the range [1, 784] with corresponding pixels X_k and X_l for a given example. We forbid input-crosses that are zero in all the train dataset. Each input-cross is not connected more than once to each hidden unit.

Forward propagation: The output of a unit, j, in the first hidden layer for the mth example, for instance, $a_{j,m}^1$, is calculated as:

$$z_{j,m}^1 = \sum_j \left(W_{ij}^1 \cdot X_i\right) + b_j^1$$

$$z_{j,m}^1 = z_{j,m}^1 - Amp_1 \cdot \frac{1}{m-1} \sum_{t=1}^{m-1} z_{j,t}^1$$

$$a_{j,m}^1 = \frac{1}{1 + e^{-z_{j,m}^1}}$$

where W_{ij}^1 is the weight from the ith input unit to the jth hidden unit, X_i is the ith input and b_j^1 is the bias induced on the jth unit in the first hidden layer. $z_{j,m}^1$ represents the field propagating from the input layer. Each time we calculate the field, $z_{j,m}^1$, we subtract the accumulative average field for the input layer of the previous $m-1$ examples, where Amp_1 is a constant representing the amplitude of reduction. Note that $z_{j,m}^1$ is not modified for m = 1.

For the second hidden layer, the output of the jth unit for the mth example, $a_{j,m}^2$, is calculated as following:

$$z_{j,m}^2 = \sum_j \left(W_{ij}^2 \cdot a_{j,m}^1\right) + b_j^2$$

$$z_{j,m}^2 = z_{j,m}^2 - Amp_2 \cdot \frac{1}{m-1} \sum_{t=1}^{m-1} z_{j,t}^2$$

$$a_{j,m}^2 = \frac{1}{1 + e^{-Z_{j,m}^2}}$$

where W_{ij}^2 is the weight from the ith unit in the first hidden layer to the jth unit in the second hidden layer, and b_j^2 is the bias induced on the jth unit in the second hidden layer. $z_{j,m}^2$ represents the field for the second layer. Each time we calculate the field, $z_{j,m}^2$, we subtract the accumulative average field for the second layer of the previous $m-1$ examples, where Amp_2 is a constant representing the amplitude of reduction. Note that $z_{j,m}^2$ is not modified for m = 1.

The output of the jth unit in the output layer, a_j^3, is calculated as following:

$$z_{j,m}^3 = \sum_j \left(W_{ij}^3 \cdot a_{j,m}^2\right) + b_j^3$$

$$a_{j,m}^3 = \frac{1}{1 + e^{-z_{j,m}^3}}$$

where W_{ij}^3 is the weight from the ith unit in the second hidden layer to the jth output unit, and b_j^3 is the bias induced on the jth output unit.

Back propagation: We use the cross entropy cost function

$$C = -\frac{1}{M}\sum_{m=1}^{M}[y_m \cdot log(a_m) + (1 - y_m) \cdot log(1 - a_m)] + \frac{\alpha}{2\eta}\sum_i W_i^2$$

where y_m stands for the desired labels and a_m stands for the current 10 output units of the output layer and η and α are constants defined in eqs. (1) and (3) in the main text, respectively. The summation is over all M training examples. The second summation is over all weights of the network. Note that for the accelerated strategy, $\eta = \eta^t$ in the above cost function.

The backpropagation method computes the gradient for each weight with respect to the cost function. The weights and biases are updated according to the advanced acceleration method[1]:

$$\eta^{t+1} = \eta^t \cdot e^{-\tau} + A_{1/2/3} \cdot \tanh\left(\beta_{1/2/3} \cdot \nabla_{W^t}C\right)$$
$$V^{t+1} = \mu \cdot V^t - |\eta^{t+1}| \cdot \nabla_{W^t}C$$
$$W^{t+1} = (1 - \alpha) \cdot W^t + V^{t+1}$$

$$\eta_b^{t+1} = \eta_b^t \cdot e^{-\tau} + A_d \cdot \tanh(\beta_d \cdot \nabla_{b^t}C)$$
$$V_b^{t+1} = \mu \cdot V_b^t - \left|\eta_b^{t+1}\right| \cdot \nabla_{b^t}C$$
$$b^{t+1} = b^t + V_b^{t+1}$$

where t is the discrete time-step, W are the weights, 1-α is a regularization constant and η is defined for each weight. A_d and β_d are constants representing the amplitude and the gain between the dth and $d+1^{th}$ layers, d=1,2 and 3. η is initialized as: $\eta_0 = A_d \cdot \tanh(\beta_d \cdot \nabla_W C_{first})$, where $\nabla_W C_{first}$ is the first computed gradient. V is initialized as: $V_0 = -|\eta_0| \cdot \nabla_W C_{first}$.

Test accuracy: The network test accuracy is calculated based on the MNIST dataset for testing, containing 10,000 input examples. The test examples are modified in the same way as the examples in the training dataset.

Optimization: The selection of the optimized parameters. For a given architecture and number of epochs, the optimization procedure first evaluates the test error over a rough grid of the adjustable parameters followed by fine-tuning grids with higher resolutions. For example, the α parameter in the range (0, 1) was first estimated under a rough grid $\Delta\alpha = 0.1$. Next, the selected range for further optimization (0, 0.1), for instance, was estimated under a resolution $\Delta\alpha = 0.01$, and finally under a resolution of $\Delta\alpha = 0.0001$. The maximal resolution was selected such that the test error for a desired resolution was unaffected by selecting a higher resolution. All other tunable parameters were optimized similarly. Note that the training error practically vanishes. For the momentum strategy and small dataset sizes, a search over the entire selected grid was possible. However, for large dataset sizes and for the acceleration strategy consists of 11 parameters an optimization of the test accuracy over a grid was beyond our computational capabilities. We note, that in order to obtain a meaningful optimization procedure, we need to average each measured point over 20-50 different samples, otherwise, the optimization procedure is dominated by stochastic fluctuations.

In cases where a complete optimization over a grid was impossible, we optimized sequentially each parameter over its grid. Nevertheless, we confirmed that a few different sequential orders of the optimized parameters result in the same optimized test accuracy and set of parameters.

The optimization is performed independently for each examined dataset size, number of examples and number of epochs. Results for the committee systems are based on the optimized selected parameters for a single system. The optimized parameters are summarized in the following tables.

We note that cross validation was confirmed using several validation databases consisting each of 10,000 random examples with the same statistics for each label as in the test set. Averaged results were in the same STD of the reported test errors. In addition, preliminary results also indicate that databases consisting of random selected examples, with different fluctuations for each label, also result in similar test errors.

APPENDIX B: FIGURE 1 – OPTIMIZED PARAMTERS

Figure 1b optimized parameters:

Momentum strategy - Parameters						
**Examples/ label**	**η**	**μ**	**α**	**Amp_1**	**Amp_2**	**Epoch**
9	0.004	0.95	0.0001	0.06	0.03	150
15	0.0095	0.65	0.00022	0.08	0.04	150
30	0.008	0.771	0.00048	0.07	0.045	150
60	0.0005	0.9555	0.0003	0.1	0.006	150

Momentum strategy - Classifications			
**Examples/ label**	**Epoch**	**Success rate**	**Std**
9	150	0.783	± 0.0244
15	150	0.8336	± 0.0114
30	150	0.8823	± 0.0076
60	150	0.916	± 0.0056

Accelerated strategy - Parameters												
**Examples/ label**	**A_1**	**A_2**	**A_3**	**β_1**	**β_2**	**β_3**	**μ**	**α**	**τ**	**Amp_1**	**Amp_2**	**Epoch**
15	0.04	0.04	0.004	1000	1000	50	0.005	0.001	0.094	0.004	0.004	50
30	0.04	0.04	0.004	1000	1000	50	0.004	0.001	0.092	0.04	0.04	30

Accelerated strategy - Classifications			
**Examples/ label**	**Epoch**	**Success rate**	**Std**
15	50	0.8391	± 0.0122
30	30	0.8854	± 0.0066

Figure 1c optimized parameters:

Momentum strategy - Classifications				
Examples/ label	Epoch	N_c Committee	Success rate committees	Std committee
9	150	101	0.8	± 0.0203
15	150	101	0.84	± 0.0115
30	150	101	0.8865	± 0.0083
60	150	101	0.919	± 0.0042

The parameters used in this figure are the same as in Figure 1b.

APPENDIX C: FIGURE 2 – OPTIMIZED PARAMTERS

Figure 2a optimized parameters:

Momentum strategy – one epoch						
Examples/ label	η	μ	α	Amp_1	Amp_2	Epoch
30	0.0043	0.955	0.0065	0.2	0.004	1
60	0.0025	0.9555	0.004	0.19	0.004	1
120	0.0021	0.95	0.0016	0.2	0.04	1
240	0.003	0.91	0.0013	0.2	0.004	1

Momentum strategy – one epoch						
Examples/ label	Epoch	N_c Committee	Success rate	Std	Success rate committees	Std committee
30	1	101	0.723	± 0.032	0.81	± 0.0082
60	1	101	0.808	± 0.0312	0.8655	± 0.0084
120	1	101	0.8583	± 0.0117	0.8996	± 0.0041
240	1	101	0.8991	± 0.0085	0.931	± 0.0015

Figure 2b optimized parameters:

Accelerated strategy – one epoch - Parameters												
**Examples/ label**	**A_1**	**A_2**	**A_3**	**β_1**	**β_2**	**β_3**	**μ**	**α**	**τ**	**Amp_1**	**Amp_2**	**Epoch**
30	0.005	0.015	0.00001	55	35	20	0.85	0.007	0.01	0.18	0.22	1
60	0.02	0.0405	0.00066	2490	3050	450	0.61	0.0046	0.054456	0.21	0.005	1
120	0.017	0.00515	0.0005	5000	2000	50	0.625	0.002	0.040822	0.205	0.0013	1
240	0.004	0.004	0.0003	1400	1800	5	0.63	0.00117	0.0095	0.005	0.002	1

Accelerated strategy – one epoch - Classifications						
**Examples/ label**	**Epoch**	**N_c Committee**	**Success rate**	**Std**	**Success rate committees**	**Std committee**
30	1	101	0.766	± 0.0189	0.815	± 0.0138
60	1	101	0.8361	± 0.0165	0.881	± 0.0119
120	1	101	0.8815	± 0.0021	0.911	± 0.0033
240	1	101	0.9142	± 0.0044	0.932	± 0.002

Figure 3b optimized parameters:

Momentum strategy - 1 hidden layers					
**Examples/ label**	**η**	**μ**	**α**	**Amp_1**	**Epoch**
30	0.005	0.945	0.007	0.15	1
60	0.0033	0.942	0.004	0.11	1
120	0.002	0.948	0.0021	0.13	1
240	0.0025	0.945	0.0008	0.003	1

**Momentum strategy - 1 hidden layers**			
**Examples/ label**	**Epoch**	**Success rate**	**Std**
30	1	0.736	± 0.0262
60	1	0.7906	± 0.0166
120	1	0.83	± 0.0135
240	1	0.857	± 0.0088

APPENDIX D: FIGURE 3 – OPTIMIZED PARAMTERS

Figure 3c optimized parameters:

**Momentum strategy - 2 hidden layers**						
**Examples/ label**	**η**	**μ**	**α**	**Amp1**	**Amp2**	**Epoch**
30	0.006	0.95	0.0066	0.15	0.005	1
60	0.0037	0.962	0.0032	0.15	0.2	1
120	0.0022	0.97	0.0015	0.15	0.2	1
240	0.002	0.97	0.00084	0.05	0.2	1

**Momentum strategy - 2 hidden layers**			
**Examples/ label**	**Epoch**	**Success rate**	**Std**
30	1	0.7013	± 0.0313
60	1	0.7717	± 0.026
120	1	0.8176	± 0.0155
240	1	0.854	± 0.012

Figure 3d optimized parameters:

**Momentum strategy - 3 hidden layers**							
**Examples/ label**	**η**	**μ**	**α**	**Amp1**	**Amp2**	**Amp3**	**Epoch**
30	0.0078	0.933	0.0046	0.34	0.14	0.13	1
60	0.0043	0.946	0.0023	0.25	0.017	0.0018	1
120	0.0046	0.943	0.001	0.47	0.12	0.9	1
240	0.003	0.945	0.0007	0.13	0.1	0.001	1

**Momentum strategy - 3 hidden layers**			
**Examples/ label**	**Epoch**	**Success rate**	**Std**
30	1	0.6328	± 0.0428
60	1	0.7164	± 0.0289
120	1	0.787	± 0.0213
240	1	0.8337	± 0.0169

APPENDIX E: FIGURE 4 – OPTIMIZED PARAMTERS

Figure 4a optimized parameters:

**Momentum strategy - 2 hidden layers**				
**Examples/ label**	**Epoch**	N_c **Committee**	**Success rate committee**	**Std committee**
30	1	101	0.8065	± 0.0077
60	1	101	0.8494	± 0.0072
120	1	101	0.8762	± 0.0043
240	1	101	0.8959	± 0.0026

The parameters used in this figure are the same as in Figure 3c.

Figure 4b optimized parameters:

Momentum strategy - 1 hidden layers					
**Examples/ label**	**η**	**μ**	**α**	**Amp_1**	**Epoch**
9	0.0006	0.945	0.0008	0.095	300
15	0.0004	0.95	0.0005	0.1	300
30	0.0054	0.978	0.00003	0.000095	300
60	0.0000089	0.9999	0.000017	0.0000975	300

Momentum strategy - 1 hidden layers			
**Examples/ label**	**Epoch**	**Success rate**	**Std**
9	300	0.7577	± 0.0202
15	300	0.802	± 0.0142
30	300	0.8533	± 0.0138
60	300	0.8869	± 0.0047

Momentum strategy - 2 hidden layers						
**Examples/ label**	**η**	**μ**	**α**	**Amp_1**	**Amp_2**	**Epoch**
9	0.00075	0.999	0.0001	0.001	0.01	300
15	0.0005	0.85	0.00007	0.01	0.004	300
30	0.0054	0.978	0.00003	0.000095	0.046	300
60	0.0000092	0.9999	0.000016	0.000098	0.047	300

Momentum strategy - 2 hidden layers			
**Examples/ label**	**Epoch**	**Success rate**	**Std**
9	300	0.7561	± 0.02
15	300	0.7988	± 0.0137
30	300	0.8546	± 0.0123
60	300	0.8875	± 0.0052

**Momentum strategy - 3 hidden layers**							
**Examples/ label**	**η**	**μ**	**α**	**Amp_1**	**Amp_2**	**Amp_3**	**Epoch**
9	0.0078	0.933	0.0046	0.34	0.14	0.13	1200
15	0.008	0.968	0.00004	0.001	0.02	0.005	500
30	0.008	0.968	0.00004	0.001	0.02	0.005	300
60	0.0065	0.971	0.00002	0.01	0.005	0.0001	600

**Momentum strategy - 3 hidden layers**			
**Examples/ label**	**Epoch**	**Success rate**	**Std**
9	1200	0.7515	± 0.0181
15	500	0.7992	± 0.0149
30	300	0.8514	± 0.0142
60	600	0.8847	± 0.0084

APPENDIX F: TWO AND THREE HIDDEN LAYERS WITH THE SAME PERFORMANCE

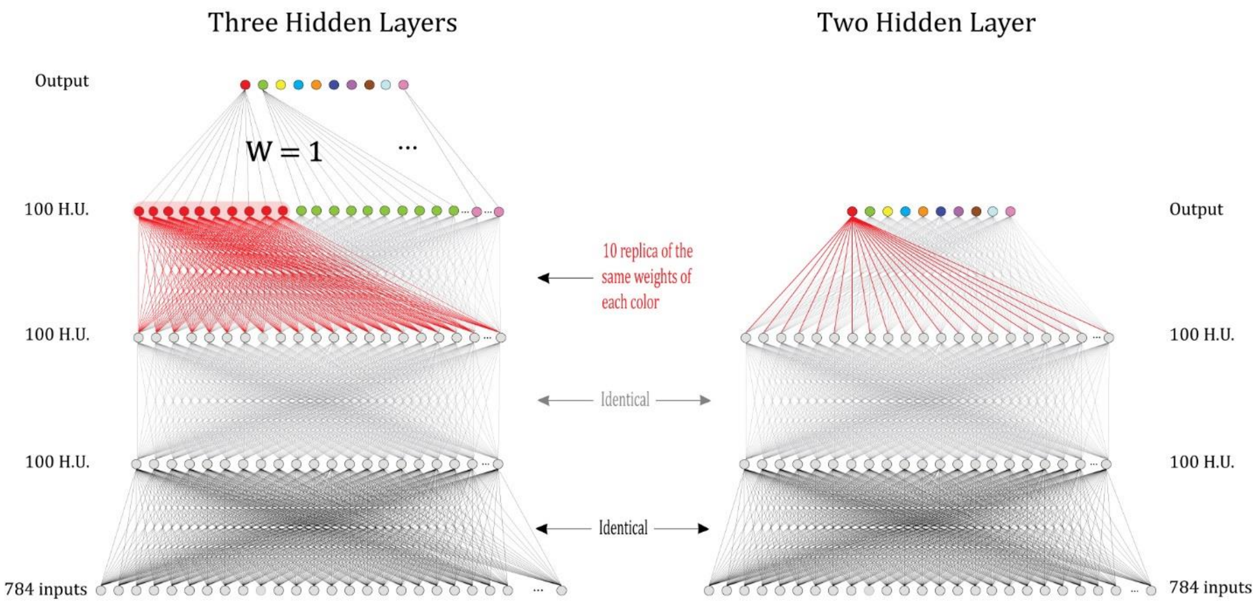

FIG. 5.

A special construction of the three hidden layers weights result in the same test error as for the same architecture but with two hidden layers only (Fig. 3a).

An example of three hidden layers neural network consisting of 784 input units, 100 hidden units for each one of the three hidden layers and 10 output units (left), and the same architecture with only two hidden layers (right). The weights between the input layer and the first hidden layer, along with the weights between the first hidden layer and the second hidden layer are identical for both networks. For the third hidden layer (left), every 10 hidden units (e.g. 10 red units) replicate the incoming weights and the output of one of the output unit (e.g. red) in the right architecture. Finally, each group of the 10 hidden units with the same color in the third hidden layer (left) are connected to a distinct output unit using a constant weight, e.g. W=1. The rest of the weights between the third hidden layer and the output layer vanish. Note that the output is different between the two networks because of the nonlinear activation function of the output units. However, the two networks make the same decision for any input, i.e. the maximal label of both networks is the same, since the nonlinear activation function is monotonically increasing.

1 Sardi, S. *et al.* Brain experiments imply adaptation mechanisms which outperform common AI learning algorithms. *Scientific Reports* **10**, 1-10 (2020).
